# Side Effects of Chloroquine and Primaquine and Symptom Reduction in Malaria Endemic Area (Mâncio Lima, Acre, Brazil)

**DOI:** 10.1155/2015/346853

**Published:** 2015-08-18

**Authors:** Cássio Braga e Braga, Antonio Camargo Martins, Athaid David Escalante Cayotopa, Wagner Werner Klein, Andreus Roberto Schlosser, Aline Ferreira da Silva, Mardelson Nery de Souza, Breno Wilson Benevides Andrade, José Alcântara Filgueira-Júnior, Wagner de Jesus Pinto, Mônica da Silva-Nunes

**Affiliations:** Centro de Ciências da Saúde e do Desporto, Universidade Federal do Acre, Campus Universitário, BR 364, Km 04, 69920-900 Rio Branco, AC, Brazil

## Abstract

Side effects of antimalarial drug can overlap with malaria symptoms. We evaluated 50 patients with *vivax* malaria in Mâncio Lima, Acre, treated with chloroquine and primaquine. Patients were evaluated for the presence of 21 symptoms before and after treatment and for reported side effects of these drugs after treatment was started. The most frequent symptoms before medication were headache, fever, chills, sweating, arthralgia, back pain, and weakness, which were present in between 40% and 76% of respondents. The treatment reduced the occurrence of these symptoms and reduced the lack of appetite, but gastrointestinal symptoms and choluria increased in frequency. There were no reports of pale stools before medication, but 12% reported the occurrence of this symptom after treatment started. Other symptoms such as blurred vision (54%), pruritus (22%), paresthesia (6%), insomnia (46%), and “stings” into the skin (22%) were reported after chloroquine was taken. The antimalarial drugs used to treat *P. vivax* malaria reduce much of the systemic and algic symptoms but cause mainly gastrointestinal side effects that may lead to lack of adherence to drug treatment. It is important to guide the patient for the appearance and the transience of such side effects in order to avoid abandoning treatment.

## 1. Introduction

Malaria is one of the main scourges of the world, affecting half a million people a year [1]. It is caused by parasites belonging to the phylum Apicomplexa, family Plasmodiidae, and genus* Plasmodium*. There are five known species that parasitize man, the most prevalent being* Plasmodium falciparum*,* P. vivax,* and* P. malariae *in tropical areas such as Latin America, the Indian subcontinent, Southeast Asia, and parts of Africa [2].* P. ovale* has been reported in Africa, Middle East, and the Indian subcontinent [3], while* P. knowlesi* has been recently described as responsible for large foci of infections in Southeast Asia [4]. The natural transmission occurs through the bite of infected female* Anopheles* mosquito,* Anopheles darlingi* being the most prevalent species in the country [5].

The Ministry of Health directs therapy and provides free antimalarial medicines used throughout the national territory, in units of the Unified Health System (SUS), through a national policy on malaria treatment. Malaria caused by* P. vivax* (or* P. ovale*) is treated with chloroquine in Brazil at a dose of 30 mg/kg divided into three days and primaquine in the 30 mg dose/day for 7 days or 15 mg/day for 14 days. Also, low doses of chloroquine can be used for the prevention of* vivax* malaria. Pregnant women and children under 6 months of age with* vivax* malaria receive only chloroquine in different dosage schedules [6].

Primaquine is the only antimalarial drug with effective activity against all the species gametocytes of* Plasmodium* which cause malaria in humans, as well as against hypnozoites, latent forms of* P. vivax* and* P. ovale* responsible for relapses [7]. However, it has hemolytic effects, especially in people with glucose-6-phosphate dehydrogenase deficiency (G6PD), in which primaquine as a stressor to the erythrocyte induces hemolysis. Hemolysis induced by primaquine can result in discoloration of urine and faeces and it is dose related [8, 9]. Gastrointestinal disorders, such as nausea, dizziness, and vomiting, are common but usually not present as severe complications, especially when primaquine is administered with food. Hypersensitivity reactions can also occur but are rare [10].

With respect to chloroquine, there are relatively few adverse effects with use in usual doses, of which the most serious are retinopathy, cardiomyopathy, myopathy, and neuromyopathy. Prolonged treatment or use of high level doses may cause retinal toxicity, long subtle symptoms of decreased visual acuity, diplopia, and bilateral loss of vision [11]. Adverse reactions of the gastrointestinal tract are the most common side effects, usually controlled by reducing drug dose. There are also reports of ototoxicity, tingling, itching, and change in skin color, in addition to seizures, insomnia, and paresthesia [12, 13].

The occurrence of symptoms specifically related to the intake of antimalarials and the overlap of symptoms of malaria with the side effects of primaquine and chloroquine may decrease adherence to treatment. Therefore, it becomes important to assess the main side effects of antimalarial drugs in patients from endemic areas, where treatment is aimed not only at improving the clinical status but also at reducing the transmission of* Plasmodium*. This study evaluated the frequency of the symptoms that commonly occur in* vivax* malaria before and after treatment and the frequency of side effects reported during the term of antimalarial treatment with chloroquine and primaquine in standardized doses according to the protocol of the Brazilian Ministry of Health [6].

## 2. Materials and Methods

### 2.1. Study Area

The study was conducted in the urban area of the municipality of Mâncio Lima, located in the Western part of the Brazilian Amazon, in the state of Acre. This municipality, with 5000 km^2^, is bordered by the cities of Cruzeiro do Sul and Rodrigues Alves and the Republic of Peru. Mâncio Lima has 14,884 inhabitants living in urban areas (57.3%), rural or riparian areas (37.9%), and Indian villages (4.8%). The county is located 38 km from Cruzeiro do Sul and 650 km from Rio Branco.

### 2.2. Study Population, Study Design, and Sampling Techniques

Patients with microscopic diagnosis of* vivax* malaria were identified in microscopy stations of the city of Mâncio Lima before the medication and invited to participate in the study. Data collection took place between May and June 2013. After signing the informed consent or having it signed by parents or legal guardians, patients answered a questionnaire on malaria symptoms that occurred up to the time of diagnosis (before antimalarial treatment), to verify the occurrence of the following symptoms: nausea, vomiting, diarrhea, pale stools, loss of appetite, mesogastric and/or hypogastric pain, pain in hypochondrium, headache, myalgia, arthralgia, back pain, retroocular pain, fever, chills, sweating, malaise or weakness, itchy throat, jaundice, choluria, dyspnea, and bitter taste sensation, covering up thereby symptoms commonly occurring in malaria and overlapping with possible side effects of chloroquine and primaquine. Between 3 and 5 days after the start of medication, a second interview was conducted asking about the occurrence of the same symptoms plus some symptoms related to the use of chloroquine that are infrequent in malaria: blurred vision, itching, stinging sensation of the skin, convulsions, numbness, and insomnia.

### 2.3. Data Analysis

The data was entered into SPSS. The distributions of relative and absolute frequencies, median, mean, and standard deviations of the variables of interest were calculated. As primaquine and chloroquine may lead to the onset of symptoms and also intensify or reduce some of the symptoms of malaria, the occurrence of symptoms before and after drug treatment was analyzed with the McNemar's test for paired samples. Some of the symptoms considered to be side effects of chloroquine and primaquine and that are not usual in malaria were assessed only after drug treatment was started.

### 2.4. Ethical Aspects

The research protocol was submitted to the Ethics Committee for Experimentation with Human Beings of Universidade Federal do Acre and approved, CAE number 22876013.9.0000.5010 (2013). Informed consent was obtained from each adult participant or legal guardian in case of minors, before the start of the study.

## 3. Results

### 3.1. Patient Characteristics

The study included 50 people with* vivax* malaria, 28 men and 22 women, aged between 7 and 68 years (average age 28.38 years). The average length of stay in an endemic area was 24.79 years, and about one-third of the study population (30%) lived in an endemic area for malaria between 11 and 20 years.

Of the 50 patients evaluated, only one had a primary infection, and all the others had had between 1 and 41 episodes of previous malaria, and 36% report having had more than 10 episodes of malaria. About 52% had an episode in the 12 months leading up to the current episode, ranging from one to 10, the number of malaria events in 2012. Most of the patients had low parasitemia (less than 200 parasites/mm^3^ in 66% of cases), 12% of them had between 301 and 500 parasites/mm^3^, and 12% were found to have 501–10000 parasites/mm^3^ (Table 1).

### 3.2. Frequency of Symptoms before and after Medication

The main symptoms observed in untreated malaria were fever (72%), chills (54%), sweating (54%), and weakness (42%), as well as pain symptoms including headache (76%), arthralgia (48%), and lower back pain (52%). Less commonly reported symptoms were retroocular pain (36%) and myalgia (30%). Gastrointestinal symptoms occurred in low frequency, such as bitter taste (30%), decreased appetite (32%), nausea (22%), vomiting (16%), and pain in hypogastric or mesogastric region (20%). Before treatment, only 20% of patients reported dark urine, and no patient reported having had pale stools.

After initiation of treatment, a significant reduction in the prevalence of fever (38%), arthralgia (26%), back pain (32%), and retroocular pain (18%) (*P* < 0.05, Table 2) was noted. However, there was significant increase in the frequency of nausea (46%), diarrhea (26%), pale stools (12%), abdominal pain (38%), bitter taste in the mouth (60%), weakness (36%), and hypochondrial pain (32%) (*P* < 0.05, Table 2). The frequency of choluria increased to 56% after initiation of antimalarial treatment (*P* = 0.01, Table 2). Other symptoms such as vomiting, loss of appetite, headache, myalgia, chills, and sweating, as well as respiratory symptoms and upper respiratory tract symptoms, suffered no major change in frequency (Table 2).

Among the adverse effects of chloroquine described in the literature and which are not often reported in malaria patients without treatment, there were reported changes in visual acuity (54%), insomnia (46%), pruritus (22%), the feeling of “stings” into the skin (22%), and paresthesias (6%). No patient reported occurrence of seizures or unconsciousness after the start of antimalarial treatment. Most gastrointestinal symptoms appeared after the patient started the antimalarial treatment (Table 3).

## 4. Discussion

The symptoms most commonly seen in patients with malaria reported in the literature are fever, chills, sweating, headache, myalgia, and arthralgia. Da Silva-Nunes and Ferreira [14] evaluated 326 cases of untreated malaria, reporting high frequency of headache (84.8%), fever (80.9%), and myalgia (68.7%), while gastrointestinal symptoms were infrequent (<20%). In pregnant women with* vivax* or* falciparum* malaria, the most frequent symptoms were also fever (97.9%), chills (71.7%), and headache (62.4%) [15].

The treatment of* vivax* malaria with chloroquine, a blood schizonticide, and primaquine (active against latent tissue forms of* P. vivax* or so-called hypnozoites) is very effective in reducing parasitemia and some of the symptoms caused by the* Plasmodium* infection, such as fever and algic symptoms. Fever tends to disappear between 24 and 48 hours after the beginning of treatment; parasitemia decreases significantly at about 48 to 72 hours, and pain symptoms decrease rapidly [16]. Pinto et al. [17] reported the disappearance of fever in 91.2% of patients, chills in 86% of patients, and headaches in 65.6% of patients at the end of the third day of treatment with chloroquine and primaquine and significant reduction of peripheral parasitemia between the second and third days of treatment.

However, chloroquine can cause side effects or intensify symptoms already present, such as abdominal discomfort, nausea, vomiting, and diarrhea. These side effects can also occur during the use of primaquine [16] being suitable to ingest the medication with food to avoid such side effects [18]. Chloroquine can rarely cause neurological symptoms, such as mental confusion, seizures, and coma, and cardiovascular symptoms such as hypotension, vasodilation, suppression of myocardial function, and cardiac arrhythmias, usually associated with rapid infusion of the drug parenterally. In usual doses for treatment of malaria, headache, blurred vision, and lichenoid rash can occur [16].

Chloroquine may block the entrance of potassium into the cells and primaquine also has an effect on blocking sodium channels. Both may also have an effect on the chloride channels present in cardiac myocytes, which may explain the occurrence of cardiac and gastrointestinal side effects [19]. In studies conducted in Belém (Pará), Silva et al. [20] found, as major side effects of chloroquine, the following symptoms: itching (5.55%), epigastric pain (1.28%), diarrhea (0.85%), and choluria (3.42%).

The itching and stinging sensation are explained by formation of haptens during the metabolism of chloroquine, which bind to degraded products erythrocytes or phospholipids involved in allergic reactions, stimulating the production of IgE-like antibodies and degranulation of mast cells and basophils. Also, the binding of chloroquine at the dermoepidermal junction may possibly result in stimulation of nerve fibers [21], causing these symptoms.

Regarding primaquine use, the most prevalent side effect is hemolytic anemia, resulting in jaundice and choluria. Weakness, malaise, and abdominal pain are also reported as primaquine side effects. Data on primaquine use worldwide indicates that, among 200 million people, there were 14 deaths in six decades related to its use, from which 12 were caused by severe hemolysis [22]. Hemolytic anemia can also occur in people without G6PD deficiency when using primaquine, although it is a very scarce event [22].

The gene encoding the G6PD is located on the X chromosome (locus Xq28), with a recessive pattern of inheritance linked to sex. It can manifest as homozygous or heterozygous inheritance in females and hemizigous in males [23]. In heterozygous females normal red blood cells can coexist with red blood cells deficient in G6PD, in variable proportion [7], and this can cause a milder hemolytic anemia when exposed to primaquine. At the same time, there are 180 different genetic variants of G6PD, resulting in different levels of hemolysis [24]. The Mediterranean variant, which predominates in Europe, Western and Central Asia, and North of India, is more severe, while the African variant (common in sub-Saharan Africa, Amazon, and Afro-Americans) is a milder variant [25]. In these milder variants, hemolysis will be clinically detected only after one or two days of exposure to primaquine [25].

The prevalence of G6PD deficiency is between 3 and 30% in endemic countries for malaria, corresponding to 350 million people [22]. In the Americas, it has a lower prevalence, varying between 5 and 10% in the Amazon region and higher levels in tropical Africa, Middle East, and the Mediterranean basin [26], such as Sudan and Congo, where it is present in more than 20% of the population [27]. In Brazil, the frequency of G6PD deficiency varies between 1.7% and 6.0%, prevailing the African variant [28]. In Manaus, a city with high levels of transmission of malaria in the Brazilian Amazon, the prevalence of G6PD was 2.5% [28]. This difference in prevalence may explain why, in Brazil, primaquine is administered to all nonpregnant patients older than 6 months with an unknown G6PD deficiency status, while in other countries with higher prevalence of this enzymatic deficiency (or in which more severe variants are common) primaquine use is very much debated and controversial [29].

The consequences of severe hemolysis are severe anemia and acute renal insufficiency. These events are dependent on the extent of hemolysis, which is related to the severity of G6PD deficiency and the dose of primaquine [26]. Recommended primaquine doses used to block* falciparum* malaria transmission (0.25 mg of base per kilogram) are usually considered to have low toxicity, because it is a single-dose treatment [26]. However, primaquine use in* vivax* malaria, aimed at preventing relapsing events, is a longer course (between 7 and 14 days), with varying dosage among malaria control policies, and therefore hemolytic events are more prone to happen [22].

In a systematic revision, Monteiro et al. [26] detected 47 cases of hemolytic anemia in patients using primaquine in Latin America and Caribbean, and 23 occurred in Brazil. However, Brazil has the largest population at risk for malaria; thus it is expected to have more cases reported. A few cases of primaquine-induced hemolysis have also been reported in other countries, such as Cuba, El Salvador, Puerto Rico, and Trinidad and Tobago, between 1963 and 2013.

In our study, the frequency of choluria (“dark urine”) increased significantly from 6% to 56% in malaria patients after primaquine was started. Since it is an event referred by the patient, we cannot be sure how many of the patients really presented with choluria, or how severe was the choluria, but it is known that G6PD deficiency occurs among Amazonian residents. Malaria and weakness also increased significantly from 42% to 64% after primaquine use. It is possible that the hemolytic anemia is the cause of weakness and malaise, reported by these patients. However, none of the patients studied suffered from severe hemolysis or severe anemia (data not shown) or required hospitalization during or after malaria treatment. Although severe events related to the use of primaquine were infrequent in our and other studies in the Amazon, it is important to bear in mind the possible adverse side effects of primaquine and also mild anemia occurring in patients with the less severe forms of G6PD deficiency, which can be associated or not with weakness. Kondrashin et al. [30] reported that subjects with no G6PD deficiency showed reduction of 1 to 2 g/dL of hemoglobin after primaquine use, while in G6PD deficient patients hemoglobin levels decreased between 3 and 5 g/dL, with fast normalization after treatment was over.

Smithuis et al. reported abdominal pain as a side effect for primaquine in* falciparum* malaria patients from Myanmar, occurring in 16% of patients receiving a single dose. There is no clear evidence why primaquine can cause abdominal pain; it can be related to the direct effect of the drug on an empty stomach [31] or related to the release of pharmacological substances during hemolysis. Despite its cause, it is noteworthy that, in our study, abdominal pain increased significantly from 6% to 32% after primaquine was started.

Although the present study has a limitation in the number of subjects studied, it was possible to detect significant association of symptoms and malaria treatment. Thus, while the treatment of* vivax* malaria with a combination of chloroquine and primaquine leads to decrease in fever and pain symptoms, it can lead to the onset of gastrointestinal symptoms and hemolysis or enhances these symptoms when already present in the patient with malaria, contributing to reducing treatment adherence, especially during the use of primaquine, which has a longer course of treatment. Moreover, the sensation of itching and stinging, although they do not occur in all patients, can be quite intense, hindering treatment with chloroquine. It is important to warn the patient of these possible side effects and seek measures to minimize them, either with concomitant use of antihistamines or antiemetics, according to the individual needs of each patient. The occurrence of severe hemolysis after primaquine use, although not common in Brazil, can still occur, and since Brazilian control programs currently do not have a policy for G6PD deficiency screening before malaria treatment is offered, health care personnel must be aware of it to interrupt primaquine use and manage its side effects properly.

## Figures and Tables

**Table 1:** Epidemiological and clinical variables of the study population.

Clinical and epidemiological characteristics (%)	
Gender	
Male	56%
Female	44%
Age	
0 to 15 years old	26%
16 to 40 years old	52%
Over 40 years old	22%
Race	
White	12%
Brown	78%
Black	6%
Indigenous	4%
Length of stay in endemic area	
0 to 10 years old	22%
11 to 20 years old	30%
21 to 30 years old	14%
31 to 40 years old	16%
More than 40 years old	18%
Malaria in previous year	
Yes	52%
No	48%
Lifelong malaria episodes	
1 to 5	36%
6 to 10	28%
More than 10	36%
Parasitemia	
<200 parasites/mm^3^	66%
200–300 parasites/mm^3^	10%
301–500 parasites/mm^3^	12%
501–10.000 parasites/mm^3^	12%

**Table 2:** Malaria symptoms occurring before and after drug treatment.

Symptoms	*N*	%	Before medication	After the start of medication	Value of *P* ^*∗*^
Gastrointestinal symptoms					
Nausea	50	44%	22%	46%	0.012
Vomiting	50	62%	16%	24%	0.481
Diarrhea	50	72%	4%	26%	0.003
Pale stools	50	88%	0%	12%	0.031
Loss of appetite	50	44%	32%	46%	0.143
Mesogastric and/or hypogastric pain	50	48%	20%	38%	0.017
Bitter taste in the mouth	50	30%	30%	60%	0.004
Pain in hypochondria	50	62%	14%	32%	0.035
Algic symptoms					
Headache	49	14%	76%	70%	0.581
Myalgia	50	58%	30%	22%	0.454
Arthralgia	50	42%	48%	26%	0.007
Lower back pain	50	40%	52%	32%	0.031
Retroocular pain	50	60%	36%	18%	0.022
General symptoms					
Fever	50	20%	72%	38%	0.001
Chills	50	34%	54%	34%	0.052
Sweating	50	36%	54%	40%	0.143
Weakness/malaise	50	22%	42%	64%	0.043
Other symptoms					
Itching throat	50	90%	4%	8%	0.625
Jaundice	50	86%	8%	14%	0.250
Choluria	50	36%	20%	56%	0.001
Dyspnea	50	84%	4%	14%	0.125

^*∗*^McNemar's test.

**Table 3:** Side effects of chloroquine and primaquine in treated patients with *vivax* malaria.

Drug	Symptom	*N*	%
Chloroquine/ primaquine	Nausea	16	32%
	Vomiting	11	22%
	Diarrhea	12	24%
	Pale stools	6	12%
	Bitter taste in the mouth	20	40%
	Pain in hypochondria	12	24%

Chloroquine	Blurred vision (3 d)	27	54%
	Pruritus (3 d)	11	22%
	“Stinging” skin (3 d)	11	22%
	Convulsion (3 d)	0	0%
	Paresthesia (3 d)	3	6%
	Insomnia (3 d)	23	46%

Primaquine	Lack of appetite	12	24%
	Meso- or hypogastric pain	16	32%
	Choluria	22	44%
	Weakness/malaise	18	36%
